# Assessment of the hepatocyte protective effects of gypenoside and its phosphorylated derivative against DHAV-1 infection on duck embryonic hepatocytes

**DOI:** 10.1186/s12917-019-1891-z

**Published:** 2019-05-07

**Authors:** Hongxu Du, Jingying Bai, Jinli Wang, Miao He, Wen Xiong, Wenjuan Yuan, Mingyu Qiao, Ke Ming, Yi Wu, Deyun Wang, Yuanliang Hu, Jiaguo Liu

**Affiliations:** Institute of Traditional Chinese Veterinary Medicine and MOE Joint International Research Laboratory of Animal Health and Food Safety, College of Veterinary Medicine, Nanjing Agricultural University, Nanjing, 210095 People’s Republic of China

**Keywords:** Duck hepatitis a virus type 1, Gypenoside, Phosphorylation modification, Hepatocyte protective effect, Apoptosis

## Abstract

**Background:**

Duck viral hepatitis (DVH) is an acute disease of young ducklings with no effective veterinary drugs for treatment. *Gynostemma pentaphyllum* is a well-known traditional Chinese medicine that plays an important role in the treatment of various diseases. Gypenoside (GP), one of the main ingredients of *Gynostemma pentaphyllum*, was reported with good hepatoprotective effects. However, its low solubility limits its application in the clinics. To improve its solubility and bioactivity, a phosphorylated derivative of gypenoside (pGP) was prepared by the sodium trimetaphosphate-sodium tripolyphosphate (STMP-STPP) method. An infrared spectroscopy method was applied to analyse the structures of GP and pGP. Then, a methyl thiazolyl tetrazolium (MTT) colorimetric assay was applied to study the hepatocyte protective efficacy of these two drugs against duck hepatitis A virus type 1 (DHAV-1) infection, and qPCR, TUNEL labelling and flow cytometry methods were used to study the relevant hepatocyte protective in vitro.

**Results:**

The infrared spectroscopy detection results showed that the phosphorylation modification of GP was successful. The MTT colorimetric assay results showed that both GP and pGP possessed good hepatocyte protective efficacy in vitro, and pGP performed better than GP when the drug was added before or after virus inoculation. Furthermore, the qPCR results revealed that both drugs could effectively inhibit the adsorption (when adding GP and pGP pre-virus inoculation), replication and release of DHAV-1, and the viral inhibition rate of pGP was greater than that of GP. The subsequent TUNEL labelling and flow cytometry assays showed that both GP and pGP could significantly inhibit duck embryo hepatocyte apoptosis induced by DHAV-1, and the inhibition effect of pGP was much stronger than that of GP.

**Conclusions:**

GP exerts good hepatocyte protective efficacy not only by inhibiting the proliferation of DHAV-1 but also by inhibiting duck embryonic hepatocyte apoptosis induced by DHAV-1, and phosphorylation modification significantly improves the antiviral and the anti-apoptotic effects of GP. Therefore, pGP has the potential to be developed into a novel drug against DHAV-1 infection.

## Background

Duck viral hepatitis (DVH) is an acute, contractible, and highly fatal infectious disease of young ducklings characterized primarily by hepatic injury [1]. Originally, the pathogens of this disease mainly include three known types of the duck hepatitis virus (DHV-1, DHV-2, and DHV-3) [2, 3]. Subsequently, DHV-1 has been classified as a member of the new genus *Avihepatovirus* in the family *Picornaviridae* and designated Duck hepatitis A virus (DHAV) according to decision of the Virus Taxonomy Ninth Report of the International Committee on Taxonomy of Viruses (ICTV) [4]. DHV-2 and DHV-3 were classified into the family *Astroviridae* and designated duck astrovirus type I (DAstV-I) and DAstV-II, respectively [5, 6]. Recently, three serotypes of DHAV have been identified: DHAV-1, the classical serotype (DHV-1); DHAV-2, a type recently isolated in Taiwan; and DHAV-3 [7], a recently described type isolated in South Korea and China [8], based on phylogenetic analysis and cross-neutralization tests [7]. Among these types, DHAV-1 is believed to be the most harmful and is distributed worldwide [9]. DHAV-1 mainly endangers young ducklings aged within 3 weeks, with the mortality rate as high as 80% or even 100%, which seriously jeopardizes the healthy development of the duck industry [10]. Although the clinical applications of an attenuated vaccine can produce a certain effect, there is still immune failure and the risk of reversion of virulence [11]. Moreover, there are no effective drugs available in the clinics so far. Therefore, the development of a new effective drug for the treatment of this disease is particularly urgent.

Traditional Chinese medicine (TCM) has been used for thousands of years and has enabled the successful many viral infectious diseases in China and some other Asian countries [12]. *Gynostemma pentaphyllum* is a famous TCM. Its main effects are heat-clearing, detoxifying and relieving cough and phlegm [13]. With the development of the chemistry and pharmacology of TCM, an increasing number of effective ingredients are being discovered. For *Gynostemma pentaphyllum*, modern pharmacology shows that its main bioactive ingredient is gypenoside (GP), which has been revealed to have hepatocyte protective, antiviral, immune-enhancing and antioxidant efficacies [14–17]. The research of Li et al. demonstrated that GP exerted its therapeutic effect on nonalcoholic steatohepatitis by regulating key transcriptional factors and lipogenic enzymes involved in fatty acid oxidation during hepatic lipogenesis [18]. Sornpet et al. also discovered that GP showed significant antiviral activity against the H5N1 virus [19]. Another study found that GP could significantly enhance T and B lymphocyte proliferation singly or synergistically with LPS and PHA [15]. However, the unfavourable properties of GP are also rather prominent, such as the low solubility, and the ease of bubble generation and haemolysis, which lowers the bioavailability of GP and its practical applications in the clinics.

To improve the biological activity of TCM ingredients, molecular modification has become an important area of pharmaceutical chemistry research in recent years. At present, the common chemical structure modification methods include sulfation [20, 21], phosphorylation [11, 22], carboxymethylation [23, 24], alkylation [25], acetylation [26], and selenization [27]. Among them, the sulfation and phosphorylation modification methods are the most common in practical applications. However, compared with sulfation modifications, phosphorylation modifications are safer, easier and more environmentally friendly [11, 22, 28]. In addition, our previous studies also found that phosphorylation of a polysaccharide is more efficacious than sulfation at increasing the DVH curative effect [22]. Therefore, in this study, GP was phosphorylated (pGP) to assess its hepatocyte protective effects and the related mechanism was also investigated.

## Results

### The infrared spectroscopy characteristics of GP and pGP

**Fig. 1:**
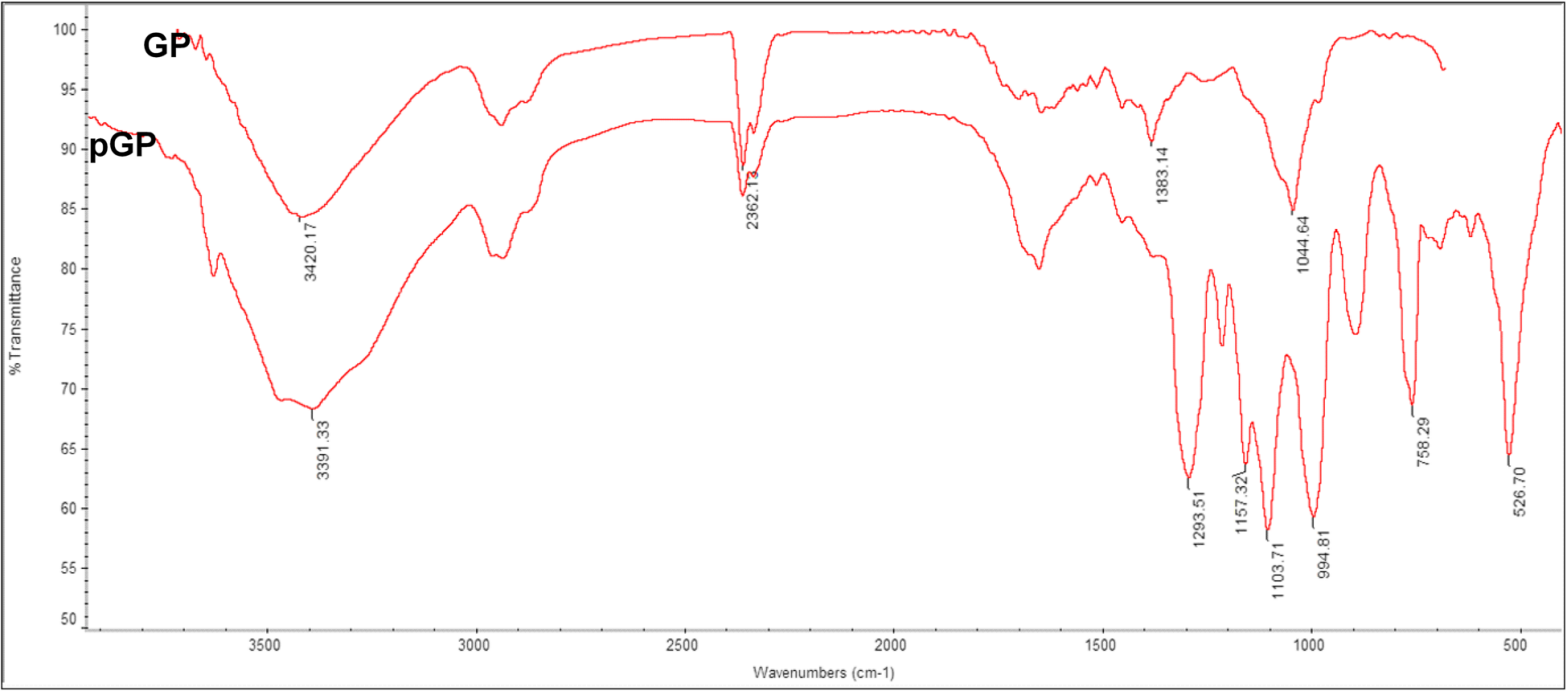
Infrared spectra of GP and pGP. Not: GP: Gypenoside; pGP: phosphorylated gypenoside

The FT-IR spectra of GP and pGP are illustrated in Fig. 1. The specific absorption bands [29, 30] of saponins were found both in GP and pGP. The absorption bands at 3600 to 3200 cm^− 1^ were attributed to the phenol hydroxyl stretching vibrations. The peaks at 1652.53 cm^− 1^ and 1383.14 cm^− 1^ were caused by the stretching vibrations of the saponin carbonyl. The absorption bands in the region of 1200 to 950 cm^− 1^ reflected C-O-C and C-O-H stretching vibrations [30]. However, with the modification, pGP had several new absorption peaks in addition to the characteristic absorption peaks of saponins. The absorption peaks at 1293.51 cm^− 1^, 994.81 cm^− 1^ and 894.06 cm^− 1^ were caused by the P=O stretching vibration, P-OH stretching vibration and P-O-C stretching vibration, respectively.

### Hepatocyte protective effects of GP and pGP on DEHs

#### GP and pGP added post-virus inoculation

Table 1 lists the *A*_570_ values and the hepatocyte protective rates of different concentrations of the two drugs on DEHs infected with DHAV-1 before the drugs were added. The results showed that the hepatocyte protective concentrations of GP ranged from 100 μg/mL to 50 μg/mL, and 100 μg/mL showed the most effective protection, with a hepatocyte protective rate of 51.91%. Moreover, all the *A*_570_ values of the pGP groups were significantly higher than those of the VC group (*p* < 0.05), and the pGP group exhibited the highest hepatocyte protective rate of 113.42% at 25 μg/mL. Moreover, the hepatocyte protective rates of GP generally increased after the phosphorylation modification.

**Table 1:** The *A*_570_ values of different treatments on DEHs when adding virus firstly (*n* = 6)

GP Concentration (μg^.^mL^− 1^)	*A*_*570*_	Hepatocyte protection rate (%)	pGP Concentration (μg^.^mL^− 1^)	*A*_570_	Hepatocyte protection rate (%)
100	0.338 ± 0.009^b^	51.91	25	0.523 ± 0.008^a^	113.42
50	0.229 ± 0.007^c^	19.94	12.5	0.486 ± 0.007^b^	97.40
25	0.167 ± 0.010^d^	1.76	6.25	0.458 ± 0.007^c^	85.28
12.5	0.174 ± 0.006^d^	3.81	3.125	0.380 ± 0.006^d^	51.52
0(VC)	0.161 ± 0.006^d^		0(VC)	0.261 ± 0.009^e^	
0(CC)	0.502 ± 0.003^a^		0(CC)	0.492 ± 0.008^a^	

^a-e^Data in same column without same superscript (a–e) differ significantly(*p* < 0.05)

*GP* Gypenoside, *pGP* phosphorylated gypenoside, *VC* virus control, *CC* cell control

#### GP and pGP added pre-virus inoculation

Table 2 shows the *A*_570_ values and the hepatocyte protective rates of different concentrations of the two drugs on DEHs infected with DHAV-1 after the drugs were added. As listed in the Table 2, the *A*_570_ values of GP at 50 to 100 μg/mL were significantly higher than those of the VC group (*p* < 0.05). The most effective concentration of GP was 100 μg/mL, with a hepatocyte protective rate of 48.17%. The *A*_570_ values of pGP were also significantly higher than those of the VC group at concentrations of 1.5625 to 25 μg/mL (*p* < 0.05). Moreover, the hepatocyte protective rates of GP generally increased after the phosphorylation modification.

**Table 2:** *A*_570_ values of different treatments when adding drug firstly (*n* = 6)

GP Concentration (μg/mL)	*A*_570_	Hepatocyte protection rate rate (%)	pGP Concentration (μg/mL)	*A*_570_	Hepatocyte protection rate rate(%)
100	0.351 ± 0.007^b^	48.17	25	0.405 ± 0.007^a^	93.55
50	0.298 ± 0.007^c^	20.42	12.5	0.306 ± 0.006^b^	40.32
25	0.274 ± 0.012^cd^	7.85	6.25	0.297 ± 0.008^b^	35.48
12.5	0.285 ± 0.007^cd^	13.61	3.125	0.276 ± 0.009^c^	24.19
6.25	0.279 ± 0.011^cd^	10.47	1.5625	0.269 ± 0.006^c^	20.43
0(VC)	0.259 ± 0.012^d^		0(VC)	0.231 ± 0.007^d^	
0(CC)	0.450 ± 0.009^a^		0(CC)	0.417 ± 0.008^a^	

^a-d^Data in same column without same superscript (a–d) differ significantly(*p* < 0.05)

*GP* Gypenoside, *pGP* phosphorylated gypenoside, *VC* virus control, *CC* cell control

#### Addition of drug and virus simultaneously

Table 3 reveals the *A*_*570*_ values and the hepatocyte protective rates of the two drugs on the DEHs infected with DHAV-1 when adding the drug and virus at the same time. As shown in the Table 3, the *A*_570_ values of GP at concentrations of 12.5 to 100 μg/mL were significantly higher than those of the VC group (*p* < 0.05), and the most effective concentration of GP was 100 μg/mL, with a hepatocyte protective rate of 108.74%. Moreover, the *A*_570_ values of pGP were significantly higher than that of the VC group at concentrations ranging from 3.125 to 25 μg/mL (*p* < 0.05), and the pGP group exhibited the highest hepatocyte protective rate of 99.26% at the concentration of 25 μg/mL. Overall, pGP showed a higher hepatocyte protective rate than GP.

**Table 3:** *A*_570_ values of different treatments when adding drug and virus simultaneously (*n* = 6)

GP Concentration (μg^.^mL^− 1^)	*A*_570_	Hepatocyte protection rate rate(%)	pGP Concentration (μg^.^mL^− 1^)	*A*_570_	Hepatocyte protection rate rate(%)
100	0.515 ± 0.004^a^	108.74	25	0.509 ± 0.003^a^	99.26
50	0.425 ± 0.008^b^	59.56	12.5	0.487 ± 0.005^a^	82.96
25	0.374 ± 0.008^c^	31.69	6.25	0.421 ± 0.007^b^	34.07
12.5	0.353 ± 0.008^d^	20.22	3.125	0.422 ± 0.010^b^	34.81
6.25	0.336 ± 0.008^de^	10.93	1.563	0.401 ± 0.007^bc^	19.26
3.125	0.322 ± 0.007^e^	3.28	0.781	0.396 ± 0.007^bc^	15.56
0(VC)	0.316 ± 0.006^e^		0(VC)	0.375 ± 0.005^c^	
0(CC)	0.499 ± 0.005^a^		0(CC)	0.510 ± 0.005^a^	

^a-e^Data in same column without same superscript (a–e) differ significantly(*P* < 0.05)

*GP* Gypenoside, *pGP* phosphorylated gypenoside, *VC* virus control, *CC* cell control

### Anti-DHAV-1 proliferation assays of GP and pGP in different phases

#### Influence of GP and pGP on DHAV-1 adsorption

**Fig. 2:**
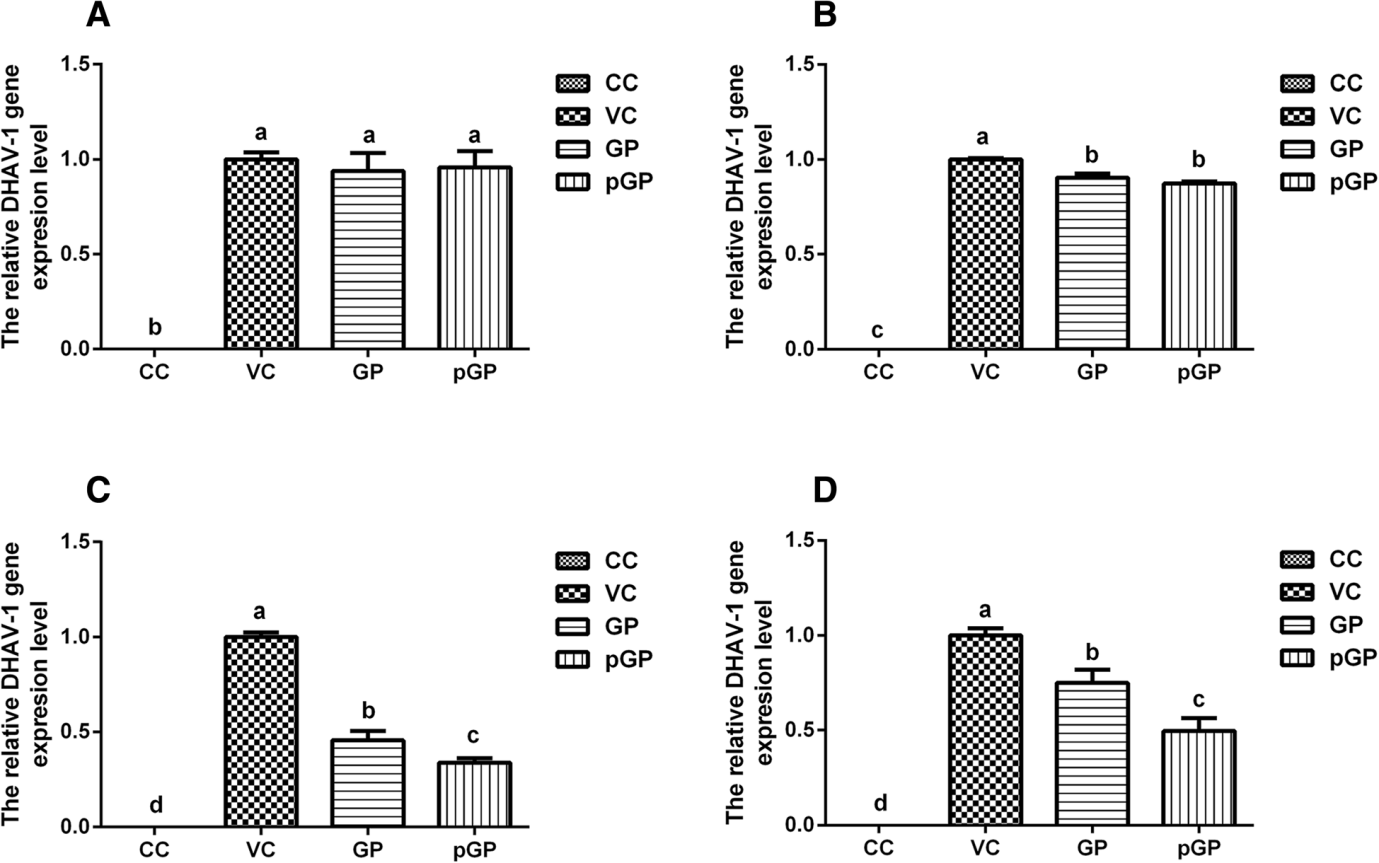
Influence of GP and pGP on the different phase of DHAV-1 proliferation (*n* = 4). Note: **a**: Adsorption (post-adding drug); **b**: Adsorption (pre-adding drug); **c**: Replication; **d**: Release. GP: Gypenoside; pGP: phosphorylated gypenoside; VC: virus control; CC: cell control. Data marked different superscript (**a**-**d**) in same figure differ significantly(*p*<0.05)

Figure 2 (a) shows the effects of GP and pGP on DHAV-1 adsorption with the post-addition manner of drug addition. There was no DHAV-1 gene expression in the CC group. In addition, the relative DHAV-1 gene expression levels in the VC, pGP and GP groups were at the same level and showed no significant differences (*p >* 0.05). Figure 2 (b) shows the effects of GP and pGP on the DHAV-1 adsorption when the drug was added pre-virus inoculation. As in Fig. 2 (a), no DHAV-1 gene expression was detected in the CC group. Moreover, with the addition of GP and pGP, the relative DHAV-1 gene expression levels in the pGP and GP groups were significantly decreased compared with those in the VC group (*p* < 0.05). In addition, the relative DHAV-1 expression levels of the GP and pGP groups were almost at the same level and had no significant difference between them (*p >* 0.05).

#### Influences of GP and pGP on DHAV-1 replication

Figure 2 (c) shows the impacts of GP and pGP on DHAV-1 replication. As shown in Fig. 2 (c), no DHAV-1 gene expression was observed in the CC group. The relative DHAV-1 gene expression levels of the GP (0.456) and pGP (0.338) groups were obviously lower than those of the VC group (*p* < 0.05). Moreover, the DHAV-1 gene expression level of the pGP group was much lower than that of the GP group (*p* < 0.05).

#### Influences of GP and pGP on DHAV-1 release

Figure 2 (d) illustrates the relative DHAV-1 gene expression levels in the CC, VC, GP and pGP groups at the DHAV-1 release phase. As shown in Fig. 2 (d), no DHAV-1 gene expression was detected in the CC group. Moreover, the relative DHAV-1 gene expression levels in the GP and pGP groups (0.749 and 0.496, respectively) were significantly lower than that of the VC group (1.000) (*p* < 0.05). Additionally, for the pGP group, the DHAV-1 gene expression level was much lower than in the GP group (*p* < 0.05).

### Effects of GP and pGP on DHAV-1-induced cell apoptosis

**Fig. 3:**
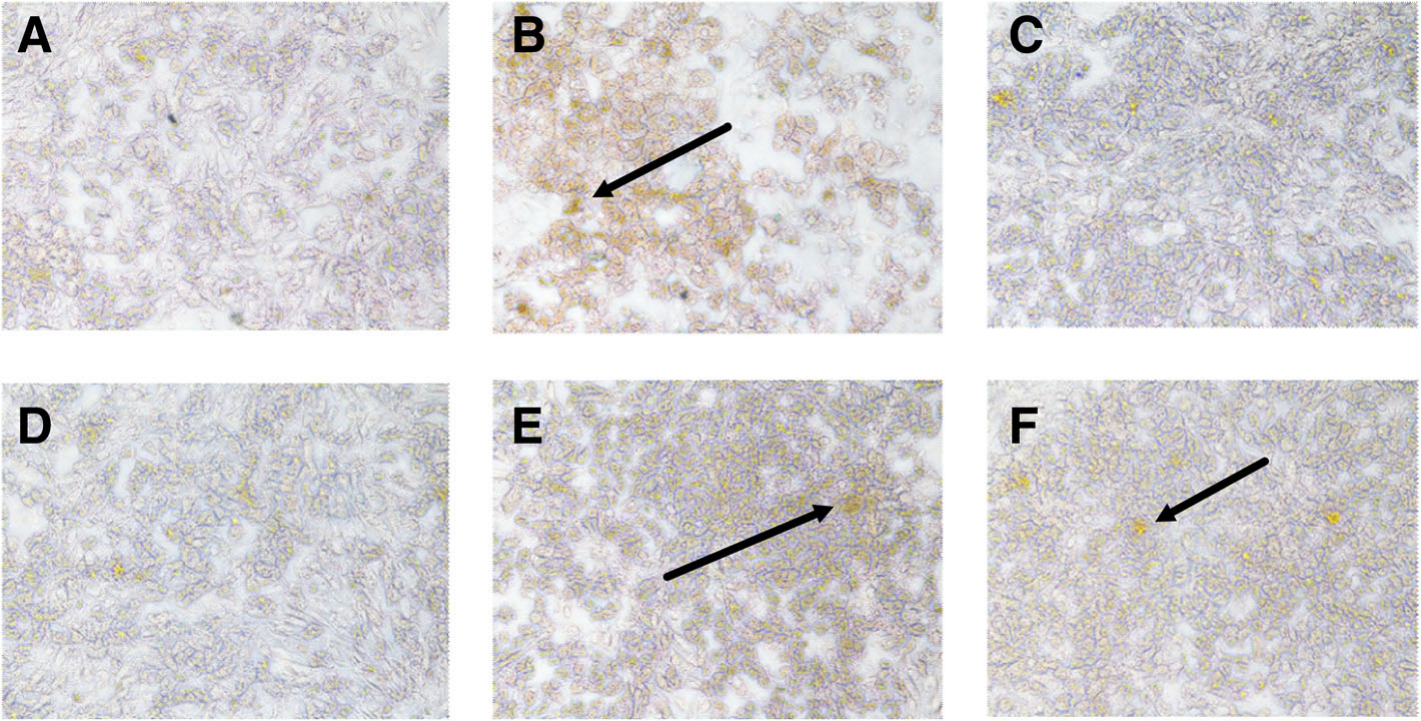
Changes of apoptotic cell levels in each group analyzed by TUNEL staining. Note: The apoptotic cells are with brown fluorescence. **a** Cell control group (CC group); **b** Virus control group (VC group); **c** Gypenoside control group (GPC group); **d** Phosphorylated gypenoside control group (pGPC) group; **e**: Gypenoside group; **f**: Phosphorylated gypenoside group

To investigate whether DHAV-1 could induce DEH apoptosis and the possible mechanisms of the hepatocyte protective effects of the GP and pGP, we conducted experiments on the normal and infected cells treated in presence or absence of GP and pGP. TUNEL staining is a detection method that can specifically bind to the 3′-OH ends of nucleic acids in apoptotic cells. Figure 3 shows the results of TUNEL staining of each group. As shown in Fig. 3, there were a few apoptotic cells (brown florescent dye) in the CC group. In the VC group, the apoptotic cell level was greatly elevated compared with that in the CC group. Compared with that in the VC group, the apoptosis-positive cell levels in the GP and pGP groups decreased greatly, and the decrease in the pGP group was more obvious. Moreover, the apoptosis-positive cell levels in the GPC and pGPC groups were almost at the same level as that in the CC group.

Subsequently, we randomly selected 5 different fields from each sample and calculated the average brown florescence rate in each group with Image-Pro Plus 6.0. As the results show in Table 4, the average brown florescence rates of CC (11.7%), GPC (12.81%) and pGPC (12.42%) were at the same level. However, the average brown florescence rate of the VC group was significantly elevated to 35.89% (*p* < 0.05). After GP or pGP treatment, the average brown florescence rate of these two groups significantly decreased 17.42 and 20.9%, respectively, which is significantly lower than that of the VC group (*p* < 0.05). Moreover, the average brown florescence rate in the pGP group (14.99%) decreased significantly (*p* < 0.05) compared with the GP group (18.47%).

**Table 4:** The average brown florescence in each group (%) (*n* = 5)

Group	Drug concentration (μg/mL)	Average brown florescence rate (%)
CC	0	11.70 ± 0.15^d^
VC	0	35.89 ± 1.07^a^
GPC	100	12.81 ± 0.25^d^
pGPC	25	12.42 ± 0.10^d^
GP	100	18.47 ± 0.21^b^
pGP	25	14.99 ± 0.43^c^

Note: Data in the same column, marked with different letters indicates a significant difference (*p* < 0.05). *CC group* Cell control group, *VC group* Virus control group, *GPC group* Gypenoside control group, *pGPC* Phosphorylated gypenoside control group, *E* Gypenoside group, *F* Phosphorylated gypenoside group

**Fig. 4:**
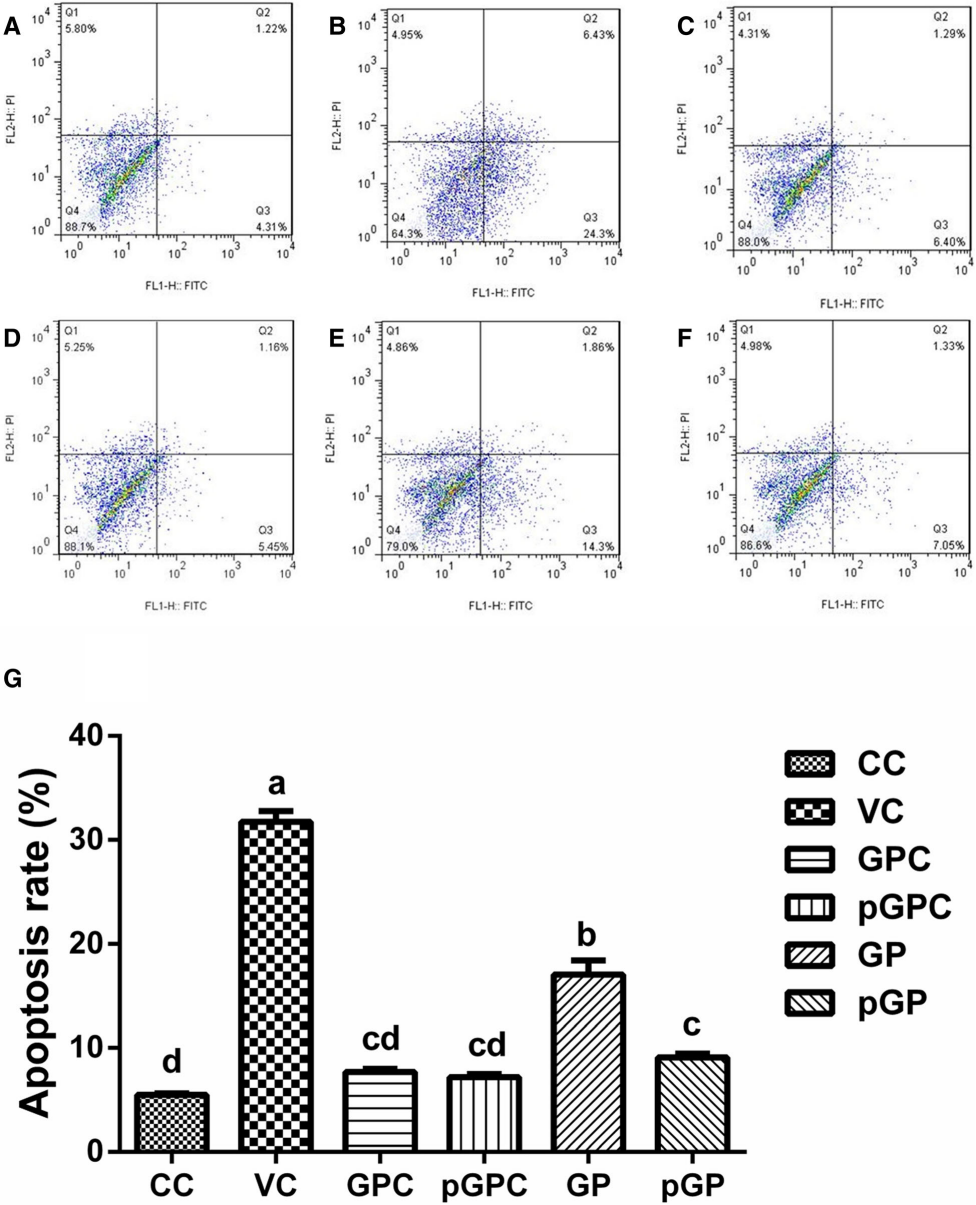
Flow cytometry detection of the effects of GP and pGP on apoptosis induced by DHAV-1 (*n* = 3). Note: **a**: Cell control group (CC group); **b** Virus control group (VC group); **c** Gypenoside control group (GPC group); **d** Phosphorylated gypenoside control group (pGPC group); **e**: GP group; **f**: pGP group. **g**: The statistical analysis results of apoptotic rate of each group. Data marked different superscript (**a**-**d**) in same figure differ significantly(*p*<0.05)

To quantitatively analyse the apoptosis rate of these different groups on DEHs, we conducted experiments on the normal and infected cells treated in the presence or absence of GP and pGP by using flow cytometry. Apoptotic cells were labelled with Annexin V-FITC, and dead cells were stained with PI. The results are presented in Fig. 4. As shown in Fig. 4 (a) and 4 (g), the apoptosis rate in the CC group was rather low, which was the lowest among these groups, and showed no significant difference between the GPC (Fig. 4 (c)) and pGPC (Fig. 4 (d)) groups (*p >* 0.05). In the VC group (Fig. 4 (b)), the apoptosis rate significantly increased to 31.75%, compared with that in the CC group (*p* < 0.05). Interestingly, both GP and pGP could reverse the increasing trend, and the apoptosis rates of the GP (Fig. 4 (e)) and pGP (Fig. 4 (f)) groups were significantly lower (14.70 and 22.67%, respectively) than that in the VC group. Moreover, compared with that of the GP group, the apoptosis rate of the pGP group decreased 7.97%, and the difference was statistically significant (*p* < 0.05).

## Discussion

The use of TCM active ingredients is a recognized shortcut for new drug development. For natural TCM ingredients, low bioactivity is a disadvantage for clinical application. Therefore, appropriate modifications are truly necessary [12]. Chemical modification of TCM ingredients is believed to be one such effective method. At present, phosphorylation of TCM ingredients is considered to be a convenient, safe and environmentally friendly modification method. In addition, our previous studies discovered that the phosphorylation modification of polysaccharides is more efficacious than the sulfation modification at increasing the DVH curative effect [22]. Therefore, in this study, we used the STMP-STPP method to modify GP.

Infrared spectroscopy is an important method to analyse and identify the molecular structure of a substance [31]. After the phosphorylation modification of GP, infrared spectroscopy was used to analyse the chemical structure of GP and its phosphorylated derivative. As the results show in Fig. 1, infrared spectroscopy analysis results showed that the peak patterns before and after the modification of GP were basically the same, indicating that the structures of GP and pGP were similar and the structure of the phosphorylated product did not change substantially. Moreover, both showed the characteristic absorption bands (1652.53 cm^− 1^, 3200⁓3650 cm^− 1^ and 2937.62 cm^− 1^) of saponins. On the other hand, several new absorption peaks were also found in the pGP spectrum (Fig. 1). Through analysis of these peaks, we found the characteristic absorption peaks of a phosphate ester (1293.51 cm^− 1^), a phosphite ester (994.81 cm^− 1^) and a pyrophosphate (894.06 cm^− 1^). All this evidence suggested that the phosphorylation modification of GP was successful.

After confirming that GP was successfully modified, we wanted to further understand the difference in the hepatocyte protective activities of GP and its phosphorylated derivative. Therefore, the MTT assay, one of the classic methods for detecting cell viability in vitro [32], was applied. The absorbance value at 570 nm reflects the viability of the cells, and the higher the *A*_*57*0_ value, the higher the cell viability [33]. As shown in Tables 1-3, the drug and virus were added in three different manners to investigate the hepatocyte protective effects of GP and pGP against DHAV-1 infection. The results showed that both GP and pGP could significantly supress the decrease in cell viability caused by DHAV-1 infection, and pGP possessed better efficacy regardless of the dosing manner. This demonstrated that both GP and pGP could exert hepatocyte protective effects against DHAV-1 infection, and with the phosphorylation modification, GP’s efficacy was greatly improved.

However, what are the mechanisms of their hepatocyte protective effects? To address this question, we investigated the indirect and direct hepatocyte protective effects of these two drugs. For the indirect effect, the above results shown in Tables 1-3 suggest that DHAV-1 infection could significantly impair DEH viability, so the antiviral activity of these two drugs may play an important role in DEH protection. For the direct effect, apoptosis is a well-known type of cell death [34], so the decrease in cell apoptosis may be another mechanism of the DEH protection efficacy of the drugs.

For the indirect hepatocyte protective (antiviral) mechanisms, we know that the proliferation stages of DHAV-1 can be generally divided into three phases: adsorption, replication and release. Therefore, we investigated the influence of GP and pGP on the adsorption phase of DHAV-1 proliferation with two different manners of drug addition. As the results presented in Fig. 2 (a, b), only when adding the drug before virus inoculation are these two drugs able to exert antiviral activity. This may indicate that both GP and pGP may block the adsorption site of DHAV-1 but cannot extricate the adsorbed virus particles. Moreover, the inhibition rates of GP and pGP were on the same level, as shown in Fig. 2 (b). These results may indicate that the blocking site of GP and pGP may be attributed to their related radical group of saponins. Subsequently, the influences of GP and pGP on the two other proliferation phases (replication and release) of DHAV-1 were studied. The results showed that both GP and pGP could inhibit the replication and release of the DHAV-1, and pGP performed better than GP. This demonstrated that the viral replication and release inhibition effects of GP were greatly improved after the phosphorylation modification.

For direct hepatocyte protective (anti-apoptotic) mechanisms, we know that apoptosis is an important type of cell death, and it not only participates in the normal physiological regulation of cells but also participates in the pathological processes of many diseases [34, 35]. Additionally, viral infection-related pathological processes have been reported to be closely related to cell apoptosis, and DHAV-1 is as well. Moreover, several studies have shown that DHAV-1 could induce cell apoptosis not only in vitro but also in vivo [36, 37]. Here, we discovered that DHAV-1 could induce cell apoptosis on DEHs. As illustrated in Fig. 3, the apoptotic cells (with brown florescence) in the VC group were greatly increased compared with those in CC group. However, the amount of brown florescence in the GP and pGP groups was less than that in the VC group. Combined with the flow cytometry detection results shown in Fig. 4, we can quantitatively review the apoptotic rate in each group. The results shown in Fig. 3 are consistent with the results shown in Fig. 4. Both of these results demonstrate that both GP and pGP could exert hepatocyte protective effects by reducing the DHAV-1-induced apoptosis of DEHs, and the phosphorylation modification of GP elevated the anti-apoptotic activity.

## Conclusion

The phosphorylation modification of GP can significantly promote its hepatocyte protective effect against DHAV-1 infection, and pGP is expected to be developed into a new drug to treat DVH induced by DHAV-1.

## Methods

### Reagents and virus

Gypenoside (GP, lot no. ZI20160922, purity ≥98%) was purchased from Nanjing Zelang Biotechnology Company (Nanjing, China). Sodium trimetaphosphate (STMP, lot no. L1226014) was purchased from Aladdin Company. Sodium tripolyphosphate (STPP, lot no. 201410711) and KBr (lot no. 20151208) were purchased from Sinopharm Group Chemical Reagent Company. Methanol (lot no. 20160410002) was purchased from Tianjin Saifurui Technology Company. The FastPure Cell/Tissue Total RNA Isolation Kit (lot no. 7E270B8), HiScript II Q RT SuperMix for qPCR (+gDNA wiper) kit (lot no. 7E220B8) and ChamQ™ SYBR qPCR Master Mix kit (lot no. 7E152L7) were purchased from Vazyme.

Dulbecco’s modified Eagle’s medium (DMEM) (Gibco, USA) containing 100 IU/mL penicillin, 100 IU/mL streptomycin, and 0.75 mg/mL glutamine was used, with 10% fetal bovine serum added as a nutritive medium and with 1% fetal bovine serum added as maintenance medium (MM). Dulbecco’s Hanks balanced salt solution (D-Hank’s) was used to wash the cells. Trypsin (Amresco, USA) was diluted to 2 mg/mL with D-Hank’s, and 3-(4,5-dimethylthiazol-2-yl)-2,5-diphenyltetrazolium bromide (MTT, Amresco, USA) was diluted to 1 mg/mL with phosphate-buffered saline (PBS, calcium and magnesium-free). DMEM, MM, D-Hank’s and MTT were filtered through 0.22 μm syringe filters and stored at 4 °C in brown or dark bottles. All other chemicals used in the experiments were of analytical grade.

Duck fertilized eggs (14-day-old) were purchased from Yangzhou Junhua Breeding Poultry Co., Ltd. The use of duck embryos in this study was approved by the Animal Ethics Committee of Nanjing Agricultural University (approval no. 2012GGC15003). The DHAV-1 (*LQ*_*2*_ strain) used in the experiment was supplied by the Shandong Institute of Poultry in China and was stored at − 80 °C. The TCID_50_ of the virus liquid was 1 × 10^− 3^/0.1 mL, determined by the Reed-Muench assay and diluted to 5 × 10^− 2^ (50 TCID_50_) with MM for the following experiments in vitro.

### Preparation of duck embryonic hepatocytes (DEHs)

The DEHs were collected according to a method described previously [38]. Briefly, the eggshell of the 14-day-old fertilized egg was thoroughly disinfected with iodophor, then the chamber end of the eggshell was knocked open with sterile forceps, the eggshell membrane was peeled off and the duck embryo was taken out from the egg. Subsequently, the liver of the duck embryo was collected and digested for 5–6 min with 0.2% trypsin to obtain DEHs. The seeding density of the DEHs was adjusted to 0.8 × 10^6^–1.2 × 10^6^/mL. Then, the cell plates were placed in a humid atmosphere of 5% CO_2_ at 37 °C for incubation. When the hepatocytes grew as a monolayer, the DEHs could be used for the following study.

### Preparation and structural identification of pGP

#### Phosphorylation modification of GP

pGP was prepared according to the STMP-STPP method described previously [11]. Briefly, 2500 mg of STMP and 1000 mg of STPP were mixed in 50 mL of double-distilled water with stirring. Thereafter, 150 mg of GP was dissolved in 50 mL of double-distilled water (by ultrasonication for 30 min), and after it was completely dissolved, 50 mL of phosphorylation reagent was added, and the mixture was stirred in a water bath at a corresponding temperature, pH and stir time. At the end of the reaction, the solution was concentrated under reduced pressure and evaporated to complete dryness. Then, the dried product was dissolved with 100% methanol and concentrated and the supernatant was dried, for 3 cycles. Finally, the dried product collected in the previous step was dissolved in the ice water and concentrated and the supernatant was collected and dried. In addition, in order to optimize the preparation process for phosphorylated GP, orthogonal tests were used. The best preparation process was as follows: 4 h reaction time, 65 °C reaction temperature, pH 9 and 5:2 STMP to STPP reaction ratio. Based on the optimal preparation process above, pGP was obtained. The contents of GP and pGP were determined using the vanillin-glacial acetic acid method [39] and the ascorbic acid method [22, 28], respectively. In addition, the results showed that the content of pGP was 99.67%, which was calculated as the sum of its GP and phosphate contents.

#### Infrared spectroscopy analysis

A Fourier transform-infrared (FI-IR) spectroscopy method was used to record the wavenumber range of GP and pGP at 4000–400 cm^− 1^ with a Nicolet 200 Magna-IR spectrometer (Nicolet Instrument Corp). In addition, OMNIC software (Nicolet Instruments Corp.) was used to analyse the major absorption peaks.

### Hepatocyte protective effects of GP and pGP against DHAV-1 infection on DEHs

According to the results of preliminary experiments, the maximum safe concentrations of GP and pGP were 100 μg/mL and 12.5 μg/mL, respectively. GP was diluted with MM from 100 to 12.5 μg/mL, and pGP was diluted from 25 to 3.125 μg/mL. The 96-well plates with DEHs were divided into cell control (CC), virus control (VC), GP and pGP groups, with duplicates of six wells for each group. The virus was diluted with MM, and GP and pGP were added to the culture plate in the following three manners [40–42]. (1) GP and pGP: The virus dilutions were added to the DEH monolayer of each group, with the exception of the CC group, at 100 μL per well. After incubation in 5% CO_2_ at 37 °C for 2 h, the virus dilutions were removed, and the plates were washed three times by using D-Hank’s. Then, different concentrations of GP and pGP dilutions were added to the DEHs of each group, with the exception of the CC and VC groups, at 100 μL per well. (2) GP and pGP addition pre-virus inoculation: The different concentrations of GP and pGP dilutions were added to the DEHs of the GP and pGP groups at 100 μL per well. After incubation in 5% CO_2_ at 37 °C for 4 h, the drug dilutions were removed, and the plates were washed three times by using D-Hank’s. Then, the virus dilutions were added to the DEHs of each group, with the exception of the CC group, at 100 μL per well. (3) Addition of drugs and virus simultaneously: DHAV-1 solutions were added to different concentrations of GP and pGP to interact for 2 h at 4 °C, and the mixed liquids were added to the DEH monolayer at 100 μL per well. The samples prepared with all three manners of drug addition were incubated in a 5% CO_2_ atmosphere at 37 °C for 96 h. Finally, the DEH cell viability was tested by the MTT method. The hepatocyte protective rate was calculated according to the formula [43]: ($\overline{A}$_*570*(drug + virus)_- $\overline{A}$_*570*(VC)_)/ ($\overline{A}$_*570*(CC)_- $\overline{A}$_*570*(VC)_) × 100%. Based on the *A*_*570*_ values and the hepatocyte protective rate, the hepatocyte protective effects of GP and pGP were analysed and compared.

### Hepatocyte protective mechanisms of GP and pGP on DEHs

#### Hepatocyte protective effects related to the antiviral mechanisms of GP and pGP

##### Virus adsorption [22, 38]

The 24-well plates with monolayer DEHs were divided into CC, VC, GP and pGP groups, with duplicates of four wells for each group. The virus was diluted with MM. GP and pGP were added to the 24-well plates according to one of the following two manners.

Drugs added post-virus inoculation: 400 μL per well of the virus dilutions were added to the monolayer of DEHs in each group (except the CC group, which was treated with 400 μL of MM per well). After incubation in 5% CO_2_ at 37 °C for 1.5 h, the virus dilutions were removed, and the cells were washed thrice with D-Hank’s. Then, 400 μL per well of the most effective concentration of the GP and pGP dilutions were added to the corresponding groups. The cells in the CC and VC groups were treated with 400 μL MM. Then, the supernatant was removed and washed three times with PBS after the cells had been incubated in an atmosphere of 5% CO_2_ at 37 °C for 1.5 h. Almost immediately, 1 mL of Trizol was added to each well. All samples were stored at − 80 °C for subsequent qRT-PCR detection.

Drugs added pre-virus inoculation: 400 μL per well of the most effective concentration of GP and pGP dilutions were added to the corresponding groups. The cells in the CC and VC groups were treated with 400 μL of MM. After incubation in an atmosphere of 5% CO_2_ at 37 °C for 6 h, the supernatant was removed, and the cells were washed thrice with D-Hank’s. Then, 400 μL per well of the virus dilutions were added to the DEH monolayer of each group (except the CC group, which was treated with 400 μL MM). Then, supernatant was removed and washed three times with PBS after the cells had been incubated in a 5% CO_2_ atmosphere at 37 °C for 1.5 h. Almost immediately, 1 mL of Trizol was added to each well. All samples were stored at − 80 °C for subsequent qRT-PCR detection.

##### Virus replication [22, 38]

The 24-well plates with the DEH monolayers were divided into CC, VC, GP and pGP groups, and four wells were duplicated for each group. Next, 400 μL per well of the virus dilutions were added into each well (except CC group, which was treated with 400 μL MM), and then incubated at 37 °C, 5% CO_2_ for 2 h. After the cells were washed three times with D-Hank’s, 400 μL per well of the most effective concentration of the GP or pGP dilution were added to the DEH monolayer of each group (except the CC and VC groups, which were treated with 400 μL of MM). The cells were incubated at 37 °C in 5% CO_2_ for 12 h. After the cells were washed three times with PBS, 1 mL of Trizol was added to each well immediately. All samples were stored at − 80 °C for subsequent qRT-PCR detection.

##### Virus release [22, 38]

The 24-well plates with DEH monolayers were divided into CC, VC, GP and pGP groups, and four wells were duplicated for each group. Next, 400 μL per well of the virus dilutions were added into each well (except CC group, which was treated with 400 μL of MM), and the plate was incubated at 37 °C in 5% CO_2_ for 30 h. Then, the cells were washed thrice with D-Hank’s, and 400 μL per well of the most effective concentration of GP or pGP was added to the cells of the corresponding groups. Cells in the CC and VC groups were treated with 400 μL of MM per well. After the cells were incubated for 2 h at 37 °C in a 5% CO_2_ incubator, 100 μL of the supernatant of each well was collected in a no-enzyme tube and mixed with 100 μL of 1.0 × 10^6^ DEHs. Then, 1 mL of Trizol was added to each tube immediately. All samples were stored at − 80 °C for subsequent qRT-PCR detection.

#### qRT-PCR detection of the relative DHAV-1 gene expression level

Total RNA was extracted from the samples mentioned above with the FastPure Cell/Tissue Total RNA Isolation Kit according to the kit instructions. Then, cDNA was synthetized using a PCR instrument (2720 Thermal Cycler PCR instrument, Applied Biosystems, USA) with the HiScript II Q RT SuperMix for qPCR (+gDNA wiper) Kit. Finally, the semi-quantitative analysis of viral replication was conducted using a real-time PCR instrument (Step One Plus™ Real Time PCR instrument, Applied Biosystems) with the ChamQ™ SYBR qPCR Master Mix Kit. The primers for DHAV-1 and β-actin were designed in our previous study. The primer sequences designed in our previous research [43] were as follows: DHAV-1 forward, 5′-GCCACCCTTCCTGAGTTTGT-3′; DHAV-1 reverse, 5′-TACCATTCCACTTCTCCTGCTT-3′; β-actin forward, 5′-CTTTCTTGGGTATGGAGTCCTG-3′; and β-actin reverse, 5′-TGATTTTCATCGTGCTGGGT-3′. The reaction parameters were as follows: 95 °C for 30 s, 95 °C for 5 s (40 cycles) and 60 °C for 30 s.

#### Hepatocyte protective effects related to the anti-apoptotic mechanisms of GP and pGP

##### Apoptosis analysis by TUNEL staining

Six-well plates with monolayer DEHs were divided into CC, VC, GP, pGP, GP control (GPC, normal cells treated with GP) and pGP control (pGPC, normal cells treated with pGP) groups, and three wells were duplicated for each group. A total of 1.5 mL per well of the virus dilution was added to each group (except the CC, GPC and pGP groups, which were treated with 1.5 mL of MM). After incubation in 5% CO_2_ at 37 °C for 2 h, the cells were washed thrice with D-Hank’s, and 1.5 mL per well of the most effective concentration of GP or pGP was added to the respective GP, GPC, PGP or pGPC groups. Meanwhile, cells in the VC and CC groups were treated with an equal volume of MM. Then, the plates were incubated in 5% CO_2_ at 37 °C for 24 h, and PBS was used as a washing buffer to wash the cells three times. After the cells were fixed by using 4% paraformaldehyde for 30 min, TUNEL staining was performed according to the instructions, and the cells were visualized by an inverted fluorescence microscope.

##### Apoptosis analysis by flow cytometry

In this experiment, cells were cultured in 6-well plates. The cell grouping and treating processes were the same as in section 2.5.2.1. After the plates were incubated for 24 h at 37 °C in a 5% CO_2_ incubator, the supernatants were collected in 1.5 mL EP tubes and centrifuged for 5 min at 300×g. The resulting supernatant was removed, and the cell pellets were collected. In addition, 1 mg/mL collagenase was added to each well of the 6-well plates to digest the DEH monolayer for 30 min at 37 °C in a 5% CO_2_ incubator. Then, the digested cells were blown off gently using disposable plastic pipettes, and the digested cells were collected in 1.5 mL EP tubes and centrifuged at 300×g for 4 min. The cell pellets were merged together, washed twice with PBS, and centrifuged at 300×g for 5 min, and the supernatants were discarded. Then, the rest of the experimental steps were performed strictly in accordance with the instructions, and the samples were detected by flow cytometry (FACSCalibur, BD).

### Statistical analysis

The relative gene expression levels were analysed with the 2^−ΔΔCT^ method [44]. All data were subjected to Duncan’s multiple range test by using SPSS 20.0 software and expressed as the mean ± S.E. Significant differences were considered *p* < 0.05.

## Abbreviations

CC: Cell control
DAstV-I: Duck astrovirus type I
DAstV-II: Duck astrovirus type II
DEHs: Duck embryonic hepatocytes
D-Hank’s: Dulbecco’s Hanks balanced salt solution
DHAV-1: Duck hepatitis A virus type 1
DHV-1: Duck hepatitis virus type 1
DMEM: Dulbecco’s modified Eagle’s medium
DVH: Duck viral hepatitis
FI-IR: Fourier transform-infrared
GP: Gypenoside
GPC: Gypenoside control
ICTV: International Committee on Taxonomy of Viruses
MM: Maintenance medium
MTT: Methyl thiazolyl tetrazolium
PBS: Phosphate-buffered saline
pGP: Phosphorylated derivative of gypenoside
pGPC: Phosphorylated derivative of gypenoside control
STMP-STPP: Sodium trimetaphosphate-sodium tripolyphosphate
TCM: Traditional Chinese medicine
TUNEL: TdT-mediated dUTP Nick-End Labelling
VC: Virus control
